# Supplementary material for: Tools for assessing child and adolescent stunting: Lookup tables, growth charts and a novel appropriate-technology “MEIRU” wallchart ‐ a diagnostic accuracy study
Source: PLOS Glob Public Health. 2023 Jul 14;3(7):e0001592. doi: 10.1371/journal.pgph.0001592 (PMC10348557; doi:10.1371/journal.pgph.0001592)
Supplement: S1 Table — (DOCX) [file pgph.0001592.s002.docx]

S1 Table: Baseline characteristics of adolescents in the study.

| Variable | Category | n (%) (n=244) | Median | IQR (SD) |
| --- | --- | --- | --- | --- |
| Age |  |  | 11.3 | 9.5 to 13.1 |
| (years:months) | 7:9 to 9:11 | 78 (32.0) |  |  |
| (y:m) | 10:0 to 11:11 | 59 (24.2) |  |  |
| (y:m) | 12:0 to 13:11 | 64 (26.2) |  |  |
| (y:m) | 14:0 to 19:0 | 43 (17.6) |  |  |
| Sex | Male | 106 (43.4) |  |  |
| HAZ |  |  | -1.6 | -2.4 to -0.9 (1.1) |
|  | Normal (HAZ≥-2) | 162 (66.4) | -1.1 | -1.6 to -0.5 (0.8) |
|  | Stunted (HAZ<-2) | 54 (22.1) | -2.5 | -2.7 to -2.2 (0.3) |
|  | Severely stunted (HAZ<-3) | 28 (11.5) | -3.5 | -3.8 to -3.3 (0.5) |
| Recruitment locations | Health centre | 4 (1.6) |  |  |
|  | Malembe | 46 (18.9) |  |  |
|  | Kirelazi | 60 (24.6) |  |  |
|  | Chikanda | 134 (54.9) |  |  |
