# Supplementary material for: Tools for assessing child and adolescent stunting: Lookup tables, growth charts and a novel appropriate-technology “MEIRU” wallchart ‐ a diagnostic accuracy study
Source: PLOS Glob Public Health. 2023 Jul 14;3(7):e0001592. doi: 10.1371/journal.pgph.0001592 (PMC10348557; doi:10.1371/journal.pgph.0001592)
Supplement: S1 Text — (DOCX) [file pgph.0001592.s003.docx]

S1 Text: Sample size calculations

Table A: Sample size calculation based on different possible misclassification rates

| **Power (1-β)** | **Type I error rate (α)** | **No event-event (p_01_)** (wrongly identified as stunted) | **Event-no event (p_10_)** (wrongly identified as not stunted) | **Sample size** |
| --- | --- | --- | --- | --- |
| **80%** | **5%** | **5%** | **1%** | **292** |
| 80% | 5% | 4% | 1% | 433 |
| 80% | 5% | 6% | 1% | 218 |
| 80% | 5% | 6% | 2% | 391 |
| 80% | 5% | 7% | 1% | 173 |
| 80% | 5% | 7% | 2% | 281 |

(Note that sample size is the same if P_01_ and p_10_ columns are reversed).

During planning phase, we used [McNemar’s χ^2^ test](https://www.statstodo.com/SSizMcNemar_Pgm.php) to determine our sample size with 80% power and type I error rate of 5%. We had to make some assumptions on the wallchart performance since it had not been used before. We have assumed that:

- 5% of the total sample would be wrongly identified as stunted by the MEIRU wallchart **(p_01_)**;
- 1% wrongly identified as not stunted **(p_10_)**.

This gives us a sample size of 292. This was also sufficient for a number of related scenarios

To account for the possibility of assumptions being incorrect, we conducted an interim analysis after 150 participants were recruited. The MEIRU wallchart performed better than anticipated:

- p_01_=2.46%;
- p_10_=0.82%.

For these figures in the same formula, a sample size of 954 participants would be required to maintain 80% power.
