# Supplementary material for: Tools for assessing child and adolescent stunting: Lookup tables, growth charts and a novel appropriate-technology “MEIRU” wallchart ‐ a diagnostic accuracy study
Source: PLOS Glob Public Health. 2023 Jul 14;3(7):e0001592. doi: 10.1371/journal.pgph.0001592 (PMC10348557; doi:10.1371/journal.pgph.0001592)
Supplement: S2 Text — (DOCX) [file pgph.0001592.s004.docx]

**S2 Text: Tables showing cross-tabulation of stunting status from HAZ and stunting status using different methods (MEIRU wallchart, WHO lookup tables, WHO growth charts)**

Table A: Cross-tabulation of stunting status from HAZ and stunting status from MEIRU wallchart.

|  | MEIRU wallchart | | | |  | 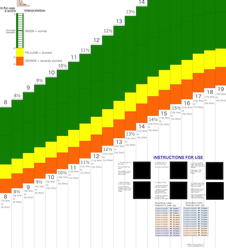 |
| --- | --- | --- | --- | --- | --- | --- |
| Gold standard HAZ | **Normal** | **Stunted** | **Severely stunted** | **Total** | |  |
| Normal | 156 | 6 | 0 | 162 | |  |
| Stunted | 2 | 50 | 2 | 54 | |  |
| Severely stunted | 0 | 1 | 27 | 28 | |  |
| Total | 158 | 57 | 29 | 244 | |  |

Shaded cells indicate concordance between the two methods.

Table B: Cross-tabulation of stunting status from HAZ and stunting status from WHO lookup tables.

|  | WHO lookup table | | |  | | 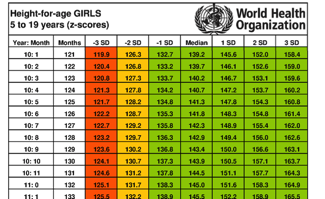 |
| --- | --- | --- | --- | --- | --- | --- |
| Gold standard HAZ | **Normal** | **Stunted** | **Severely stunted** | | **Total** |  |
| Normal | 100 | 46 | 16 | | 162 |  |
| Stunted | 1 | 17 | 36 | | 54 |  |
| Severely stunted | 0 | 0 | 28 | | 28 |  |
| Total | 101 | 63 | 80 | | 244 |  |

Shaded cells indicate concordance between the two methods.

Table C: Cross-tabulation of stunting status from HAZ and stunting status from WHO growth charts.

|  | WHO growth chart | | |  | | 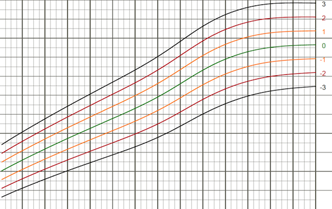 |
| --- | --- | --- | --- | --- | --- | --- |
| Gold standard HAZ | **Normal** | **Stunted** | **Severely stunted** | | **Total** |  |
| Normal | 99 | 27 | 13 | | 139 |  |
| Stunted | 16 | 17 | 16 | | 49 |  |
| Severely stunted | 7 | 3 | 17 | | 27 |  |
| Total | 122 | 47 | 46 | | 215 |  |

Shaded cells indicate concordance between the two methods.
