# Supplementary material for: Tools for assessing child and adolescent stunting: Lookup tables, growth charts and a novel appropriate-technology “MEIRU” wallchart ‐ a diagnostic accuracy study
Source: PLOS Glob Public Health. 2023 Jul 14;3(7):e0001592. doi: 10.1371/journal.pgph.0001592 (PMC10348557; doi:10.1371/journal.pgph.0001592)
Supplement: S3 Text — (DOCX) [file pgph.0001592.s005.docx]

**S3 Text: Diagnostic accuracies of each test method, by HSA**

Table A: Diagnostic accuracies of the MEIRU wallchart around the HAZ-2 cut off, by HSA.

| HSA | Sens, % | Spec, % | PPV, % | NPV, % | Accuracy |
| --- | --- | --- | --- | --- | --- |
| 1 (n=3)* | - | - | - | - | - |
| 2 (n=29) | 92.3 | 93.8 | 92.3 | 93.8 | 93.1 |
| 3 (n=25) | 100 | 100 | 100 | 100 | 100 |
| 4 (n=21) | 100 | 100 | 100 | 100 | 100 |
| 5 (n=25) | 100 | 100 | 100 | 100 | 100 |
| 6 (n=15)* | - | - | - | - | - |
| 7 (n=20) | 100 | 100 | 100 | 100 | 100 |
| 8 (n=27) | 100 | 90.9 | 94.1 | 100 | 96.3 |
| 9 (n=11) | 100 | 100 | 100 | 100 | 100 |
| 10 (n=18) | 100 | 92.9 | 93.3 | 100 | 96.4 |
| 11 (n=20) | 100 | 94.4 | 66.7 | 100 | 95.0 |
| 12 (n=20) | 66.7 | 94.1 | 66.7 | 94.1 | 90.0 |

Table B: Diagnostic accuracies of the WHO lookup table around the HAZ-2 cut-off, by HSA.

| HSA | Sens, % | Spec, % | PPV, % | NPV, % | Accuracy |
| --- | --- | --- | --- | --- | --- |
| 1 (n=3)* | - | - | - | - | - |
| 2 (n=29) | 100 | 62.5 | 68.4 | 100 | 79.3 |
| 3 (n=25) | 88.9 | 68.8 | 61.5 | 91.7 | 76.0 |
| 4 (n=21) | 100 | 93.3 | 85.7 | 100 | 95.2 |
| 5 (n=25) | 100 | 60 | 62.5 | 100 | 76.0 |
| 6 (n=15)* | - | - | - | - | - |
| 7 (n=20) | 100 | 82.4 | 50 | 100 | 85.0 |
| 8 (n=27) | 100 | 36.4 | 69.6 | 100 | 74.1 |
| 9 (n=11)* | - | - | - | - | - |
| 10 (n=18) | 100 | 28.6 | 58.3 | 100 | 64.3 |
| 11 (n=20) | 100 | 50 | 18.2 | 100 | 55.0 |
| 12 (n=20) | 100 | 94.1 | 75 | 100 | 95.0 |

Table C: Diagnostic accuracies of WHO growth charts around the HAZ-2 cut-off, by HSA.

| HSA | Sens, % | Spec, % | PPV, % | NPV, % | Accuracy |
| --- | --- | --- | --- | --- | --- |
| 1 (n=3)* | - | - | - | - | - |
| 2 (n=29) | 100 | 62.5 | 68.4 | 100 | 79.3 |
| 3 (n=25) | 66.7 | 100 | 100 | 84.2 | 88.0 |
| 4 (n=21) | 100 | 93.3 | 85.7 | 100 | 95.2 |
| 5 (n=25) | 50 | 93.3 | 83.3 | 73.7 | 76.0 |
| 6 (n=15)* | - | - | - | - | - |
| 7 (n=20) | 100 | 52.9 | 27.3 | 100 | 70.6 |
| 8 (n=27) | 18.8 | 63.6 | 42.9 | 35 | 37.0 |
| 9 (n=11)* | - | - | - | - | - |
| 10 (n=18) | 85.7 | 57.1 | 66.7 | 80.0 | 71.4 |
| 11 (n=20) | 100 | 38.9 | 15.4 | 100 | 45.0 |
| 12 (n=20) | 100 | 82.4 | 50 | 100 | 85.0 |

*Diagnostic performance markers could not be calculated due to insufficient data points.
