# Supplementary material for: Tools for assessing child and adolescent stunting: Lookup tables, growth charts and a novel appropriate-technology “MEIRU” wallchart ‐ a diagnostic accuracy study
Source: PLOS Glob Public Health. 2023 Jul 14;3(7):e0001592. doi: 10.1371/journal.pgph.0001592 (PMC10348557; doi:10.1371/journal.pgph.0001592)
Supplement: S4 Text — (DOCX) [file pgph.0001592.s006.docx]

**S4 Text: Questionnaire responses on speed of each method and individual preferences**

**Fig A: Questionnaire responses on speed of each method**

**
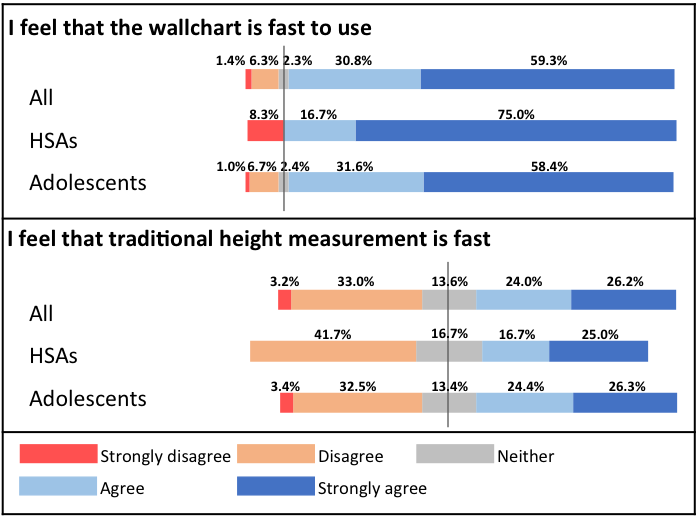
**

**Fig B: Questionnaire responses on how participants liked each method**

**
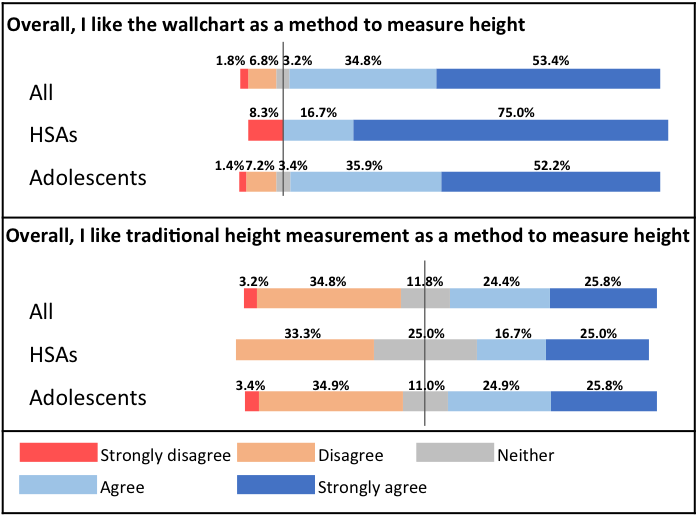
**
