# Supplementary material for: Tools for assessing child and adolescent stunting: Lookup tables, growth charts and a novel appropriate-technology “MEIRU” wallchart ‐ a diagnostic accuracy study
Source: PLOS Glob Public Health. 2023 Jul 14;3(7):e0001592. doi: 10.1371/journal.pgph.0001592 (PMC10348557; doi:10.1371/journal.pgph.0001592)

**S5 Text: Questionnaire responses on perception of stunting in the community and individual views on stunting**

**Fig A: Perception of importance of stunting in the community**


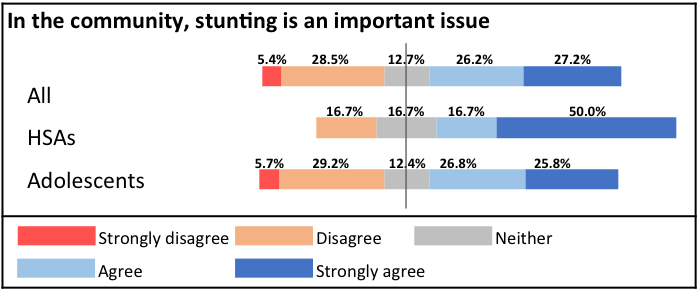


**Fig B: How worried the individual would be if they were stunted**


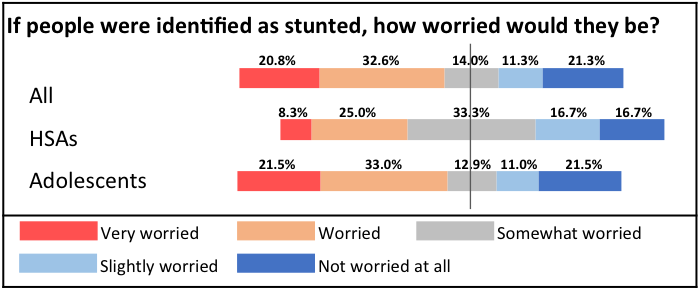


**Fig C: How embarrassed the individual would be if they were stunted**


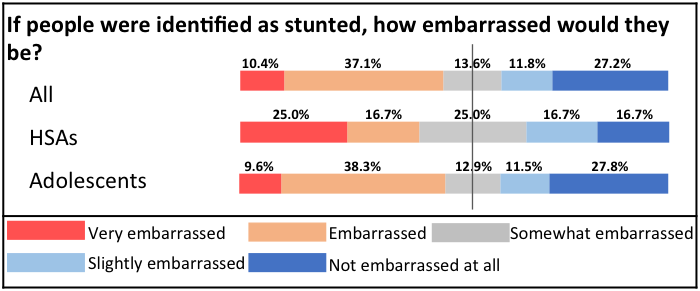

Supplement: S5 Text — (DOCX) [file pgph.0001592.s007.docx]
