# Supplementary material for: Tools for assessing child and adolescent stunting: Lookup tables, growth charts and a novel appropriate-technology “MEIRU” wallchart ‐ a diagnostic accuracy study
Source: PLOS Glob Public Health. 2023 Jul 14;3(7):e0001592. doi: 10.1371/journal.pgph.0001592 (PMC10348557; doi:10.1371/journal.pgph.0001592)
Supplement: Attachment — Submitted filename: Rebuttal letter.pdf [file pgph.0001592.s009.pdf]

Rebuttal letter

Manuscript title: Tools for assessing child and adolescent stunting: Lookup tables, growth charts and a novel appropriate-technology “MEIRU” wallchart - a diagnostic accuracy study

Thank you for the opportunity to address the comments from the Editors and Reviewers. The authors have taken all your feedback on board and hope that the Editors and Reviewers will be satisfied with the further amendments which we have made to the manuscript.

Journal Requirements:

1. Please include a complete copy of PLOS’ questionnaire on inclusivity in global research in your revised manuscript. Our policy for research in this area aims to improve transparency in the reporting of research performed outside of researchers’ own country or community. The policy applies to researchers who have travelled to a different country to conduct research, research with Indigenous populations or their lands, and research on cultural artefacts. The questionnaire can also be requested at the journal’s discretion for any other submissions, even if these conditions are not met. Please find more information on the policy and a link to download a blank copy of the questionnaire here: https://journals.plos.org/plosone/s/best-practices-in-research-reporting. Please upload a completed version of your questionnaire as Supporting Information when you resubmit your manuscript.

Response: This has now been uploaded as S17, which has also been referenced on page 8.

2. Please amend your detailed Financial Disclosure statement. This is published with the article. It must therefore be completed in full sentences and contain the exact wording you wish to be published.

a) Please clarify all sources of financial support for your study. List the grants, grant numbers, and organizations that funded your study, including funding received from your institution. Please note that suppliers of material support, including research materials, should be recognized in the Acknowledgements section rather than in the Financial Disclosure.
b) State the initials, alongside each funding source, of each author to receive each grant. For example: "This work was supported by the National Institutes of Health (####### to AM; ###### to CJ) and the National Science Foundation (###### to AM)."
c) State what role the funders took in the study. If the funders had no role in your study, please state: “The funders had no role in study design, data collection and analysis, decision to publish, or preparation of the manuscript.”
d) If any authors received a salary from any of your funders, please state which authors and which funders.

Response: Please see amended Financial Disclosure statement.

3. Please update your online Competing Interests statement. If you have no competing interests to declare, please state: “The authors have declared that no competing interests exist.”

Response: Please see amended Competing Interests statement.

4. Some material included in your submission may be copyrighted. According to PLOS’s copyright policy, authors who use figures or other material (e.g., graphics, clipart, maps) from another author or copyright holder must demonstrate or obtain permission to publish this material under the Creative Commons Attribution 4.0 International (CC BY 4.0) License used by PLOS journals. Please closely review the details of PLOS’s copyright requirements here: PLOS Licenses and Copyright. If you need to request permissions from a copyright holder, you may use PLOS's Copyright Content Permission form.

Please respond directly to this email or email the journal office and provide any known details concerning your material's license terms and permissions required for reuse, even if you have not yet obtained copyright permissions or are unsure of your material's copyright compatibility.

Potential Copyright Issues:

Figure 1: Please confirm whether you drew the images / clip-art within the figure panels by hand. If you did not draw the images, please provide (a) a link to the source of the images or icons and their license / terms of use; or (b) written permission from the copyright holder to publish the images or icons under our CC-BY 4.0 license. Alternatively, you may replace the images with open source alternatives. See these open source resources you may use to replace images / clip-art:
- https://commons.wikimedia.org
- https://openclipart.org/"

Figures 4 and 5 contain branding/a logo. We are not permitted to publish this under our CC-BY 4.0 license, even with permission. We ask that you please remove or replace it.

Response: We can confirm that Figure 1 is original with images drawn or photographed by the authors. We have updated Figures 4 and 5 to remove the logo.

5. Please review your reference list to ensure that it is complete and correct. If you have cited papers that have been retracted, please include the rationale for doing so in the manuscript text, or remove these references and replace them with relevant current references. Any changes to the reference list should be mentioned in the rebuttal letter that accompanies your revised manuscript. If you need to cite a retracted article, indicate the article's retracted status in the References list and also include a citation and full reference for the retraction notice.

Response: Please see amendments on reference list.

Additional Editor Comments (if provided):

Thank you for submitting your work to PGPH for publication. It has received favourable reviews from two independent reviewers. I suggest you carefully address their comments and resubmit for consideration.

I have some minor issues I would like you to address:

1. Multiple in-text references should be in one parenthesis, e.g. [15, 25, 45]. In case references are in sequence, e.g. 24,25,26,27,28,29 should be written as [24-29].
2. Include standard deviation (SD) in the binary form of the z-scores. e.g. HAZ-2SD etc. This correction should be made throughout the manuscript.

Response:
1. We have amended the in-text references as mentioned.
2. We have added SD for HAZ at different cut-offs in the S3 Table showing baseline characteristics of adolescents in this study.

Good luck in the revision.

[Note: HTML markup is below. Please do not edit.]

Reviewers' comments:

Reviewer's Responses to Questions

Comments to the Author

1. Does this manuscript meet PLOS Global Public Health's publication criteria? Is the manuscript technically sound, and do the data support the conclusions? The manuscript must describe methodologically and ethically rigorous research with conclusions that are appropriately drawn based on the data presented.

Reviewer #1: Yes

Reviewer #2: Yes

---

2. Has the statistical analysis been performed appropriately and rigorously?

Reviewer #1: Yes

Reviewer #2: Yes

---

3. Have the authors made all data underlying the findings in their manuscript fully available (please refer to the Data Availability Statement at the start of the manuscript PDF file)?

The PLOS Data policy requires authors to make all data underlying the findings described in their manuscript fully available without restriction, with rare exception. The data should be provided as part of the manuscript or its supporting information, or deposited to a public repository. For example, in addition to summary statistics, the data points behind means, medians and variance measures should be available. If there are restrictions on publicly sharing data—e.g. participant privacy or use of data from a third party—those must be specified.

Reviewer #1: No

Reviewer #2: Yes

Response: Please find all data underlying our findings uploaded as Supporting Information file S16.

---

4. Is the manuscript presented in an intelligible fashion and written in standard English?

PLOS Global Public Health does not copyedit accepted manuscripts, so the language in submitted articles must be clear, correct, and unambiguous. Any typographical or grammatical errors should be corrected at revision, so please note any specific errors here.

Reviewer #1: Yes

Reviewer #2: Yes

---

5. Review Comments to the Author

Please use the space provided to explain your answers to the questions above. You may also include additional comments for the author, including concerns about dual publication, research ethics, or publication ethics. (Please upload your review as an attachment if it exceeds 20,000 characters)

Reviewer #1: This is a good attempt at introducing innovation to the measurement of child health outcomes. The advantages of the proposed methods over the traditional ones should be relevant for policy especially in dealing with the lags issues between the publication of demography surveys. However, the external validity of the proposed method could be improved upon if consistent results are obtained across: developing countries; and other child health outcomes (wasting, underweight). Are findings sensitive to context? Also, it is unclear on which theoretical framework is the methodology for measurement based? can the study be more clear on how guidance are followed in the development of the measurement index?

Response:

Thank you for your comments. Stunting is a reflection of chronic malnutrition, whereas other anthropometric measurements such as wasting is more reflective of malnutrition over a shorter period of time. Therefore, identifying children with stunting will not necessarily identify children with wasting.

The reference standard for measurement of HAZ (and therefore stunting status) was used following the methods outlined by WHO, please find explanation of this method on page 10. We developed the MEIRU chart to be a method that is effective, well-accepted, and cost-effective, based on the Wilson-Jungner screening test criteria (1).

Reviewer #2: First I congratulate the authors for coming up with this innovative work of method to assess the stunting o children and adolescent groups. The manuscript is well written, data is well presented however, there few comments which need to be addressed as per attachment.
Reviewer Comments:
LINE DESCRIPTION
66 Remove repeated word :… We recruited 244 recruited.
235 It is good to used simple available materials.., however I doubt if the use of canvas held by two wooden sticks is stable enough for accurate readings. Will you also suggest printing on a soft board for stability and for accurate readings?
240 and 242 Do they stand in the same manner as in stadiometer/length board?, i.e., do the same rules apply?
318 You mentioned only data for males, can you also mention the data girls as well?.
326 244 is your total sample, if so, I think you should instead of n=244 it should read (N=244)
422 Insert space after 41.1
425 Recheck for punctuation (full stop) between adolescents and In combination…
411 and 442 Indicate what solutions to be taken as it is highly needed to minimize these errors in order to give MEIRU more value.
462 May be you need to Rephrase the sentence by adding the word approach. “In routine clinical practice where this approach is not used, “
470 and 471 Rephrase the sentence to read: “For instance, by not positioning the chart properly on the floor could lead to either under- or over-reading of heights”.
472 I agree to have it painted on the wall or on a soft board for stability among other factors! In future what about a child <2yrs who cannot stand upright? Considering in surveys and clinics all children need to be attended also for their stunting status. Link with sentence in line 488-490
504 i) Contextualizing (translating into a local situation) proved frameworks like UNICEF conceptual framework for malnutrition can ease the burden.
ii) there should be an accepted treatment recognized for the disease: Note: since nutritional stunting is not a disease rather it is a condition; then I suggest you rephrase the sentence. ..”there should an accepted approach/strategy to correct the stunting condition”
Otherwise this work is a good innovation! With modifications/improvements it can be scaled-up to replace the traditional methods especially at community level.

Response:

Thank you for your comments. We have made the phrasing and formatting amendments following your suggestions.

235 We acknowledge that there is potential for error given the use of a canvas chart held by wooden poles. This was done to ensure portability of the MEIRU chart. A soft board would indeed provide more stability and accuracy for longer-term use.
240 and 242 Yes participants stand in the same manner as a stadiometer, however as the MEIRU wallchart does not come with an attached horizontal headpiece, we have improvised by using a book as a makeshift carpenter’s square to ensure that readings were accurate.
441 and 442 The errors mentioned here apply to the methods currently used in clinical practice – the WHO lookup tables and WHO growth charts. Therefore a simpler method like the MEIRU chart will help to address and minimise these errors.

---

6. PLOS authors have the option to publish the peer review history of their article (what does this mean?). If published, this will include your full peer review and any attached files.

Do you want your identity to be public for this peer review? If you choose “no”, your identity will remain anonymous but your review may still be made public.

For information about this choice, including consent withdrawal, please see our Privacy Policy.

Reviewer #1: No

Reviewer #2: No

References:

1. Wilson JMG, Jungner G. Principles and practice of screening for disease. World Health Organization, 1968.
